# Predictors of Rifampicin-Resistant Tuberculosis Mortality among HIV-Coinfected Patients in Rwanda

**DOI:** 10.4269/ajtmh.20-1361

**Published:** 2021-05-17

**Authors:** Dominique Savio Habimana, Jean Claude Semuto Ngabonziza, Patrick Migambi, Yves Mucyo-Habimana, Grace Mutembayire, Francine Byukusenge, Innocent Habiyambere, Eric Remera, Placidie Mugwaneza, Ivan Emil Mwikarago, Jean Baptiste Mazarati, Innocent Turate, Sabin Nsanzimana, Tom Decroo, Catherine Bouke de Jong

**Affiliations:** 1 HIV, AIDS, STIs and Other Blood Borne Infections Division, Institute of HIV/AIDS Disease Prevention and Control, Rwanda Biomedical Centre, Kigali, Rwanda;; 2 National Reference Laboratory Division, Department of Biomedical Services, Rwanda Biomedical Center, Kigali, Rwanda;; 3 Mycobacteriology Unit, Department of Biomedical Sciences, Institute of Tropical Medicine, Antwerp, Belgium;; 4 Department of Biomedical Sciences, University of Antwerp, Antwerp, Belgium;; 5 Tuberculosis and Other Respiratory Diseases Division, Institute of HIV/AIDS Disease Prevention and Control, Rwanda Biomedical Center, Kigali, Rwanda;; 6 Department of Biomedical Services, Rwanda Biomedical Center, Kigali, Rwanda;; 7 Department of Institute of HIV/AIDS Disease Prevention and Control, Rwanda Biomedical Centre, Kigali, Rwanda;; 8 Rwanda Biomedical Centre, Kigali, Rwanda;; 9 Department of Clinical Sciences, Institute of Tropical Medicine, Antwerp, Belgium;; 10 Research Foundation Flanders, Brussels, Belgium

## Abstract

Tuberculosis (TB), including multidrug-resistant (MDR; i.e., resistant to at least rifampicin and isoniazid)/rifampicin-resistant (MDR/RR) TB, is the most important opportunistic infection among people living with HIV (PLHIV). In 2005, Rwanda launched the programmatic management of MDR/RR-TB. The shorter MDR/RR-TB treatment regimen (STR) has been implemented since 2014. We analyzed predictors of MDR/RR-TB mortality, including the effect of using the STR overall and among PLHIV. This retrospective study included data from patients diagnosed with RR-TB in Rwanda between July 2005 and December 2018. Multivariable logistic regression was used to assess predictors of mortality. Of 898 registered MDR/RR-TB patients, 861 (95.9%) were included in this analysis, of whom 360 (41.8%) were HIV coinfected. Overall, 86 (10%) patients died during MDR/RR-TB treatment. Mortality was higher among HIV-coinfected compared with HIV-negative TB patients (13.3% versus 7.6%). Among HIV-coinfected patients, patients aged ≥ 55 years (adjusted odds ratio = 5.89) and those with CD4 count ≤ 100 cells/mm^3^ (adjusted odds ratio = 3.77) had a higher likelihood of dying. Using either the standardized longer MDR/RR-TB treatment regimen or the STR was not correlated with mortality overall or among PLHIV. The STR was as effective as the long MDR/RR-TB regimen. In conclusion, older age and advanced HIV disease were strong predictors of MDR/RR-TB mortality. Therefore, special care for elderly and HIV-coinfected patients with ≤ 100 CD4 cells/mL might further reduce MDR/RR-TB mortality.

## INTRODUCTION

In 2019, tuberculosis (TB) was ranked among the 10 leading causes of death worldwide, with approximately 1.4 million people dying of TB.^1^ Rifampicin is the most potent first-line anti-TB drug. In 2019, approximately half a million patients worldwide developed rifampicin-resistant tuberculosis (RR-TB), of whom 78% had concomitant resistance to isoniazid, thus multidrug-resistant (MDR) TB.^1^ Of all MDR/RR-TB cases, only 44% were notified, and only 38% enrolled on MDR/RR-TB treatment.^1^ Treatment of RR-TB is not different from treatment of MDR-TB.^1^ MDR/RR-TB treatment success was as low as 56%.^1^ Furthermore, people living with HIV (PLHIV) had 19 times higher risk of developing TB than those without, and 8.6% of all incident TB cases were among PLHIV.^1^

Rwanda, like many sub-Saharan Africa countries, has a generalized HIV epidemic. The HIV prevalence in Rwanda is 3%.^2^ From July 2018 to June 2019, 21% of all incident TB, including MDR/RR-TB, developed among PLHIV,^3^ whereas approximately 40% of all MDR/RR-TB patients were HIV-coinfected.^4^

In 2005, Rwanda launched the programmatic management of MDR/RR-TB (PMDT), including surveillance, notification, detection, and treatment of MDR/RR-TB.^5^ Increasingly, the diagnosis of MDR/RR-TB relied on rapid molecular tests, such as Xpert MTB/RIF.^4^ In 2014, the shorter MDR/RR-TB treatment regimen (STR) was implemented.^6^ A recently published retrospective study showed that the reduction of diagnostic and therapeutic delays was associated with a reduced mortality over time among patients diagnosed with MDR/RR-TB between 2005 and 2016.^4^ The same study showed that HIV-coinfected patients, although having less MDR/RR-TB diagnostic and therapeutic delay compared with HIV-negative patients, had 2 times higher odds of dying.^4^ Another Rwandan study confirmed that HIV-coinfected patients were more at risk of having an unfavorable treatment outcome.^7^ However, predictors of adverse outcomes among HIV-coinfected patients on MDR/RR-TB treatment had not yet been assessed.

Evidence on STR outcomes among PLHIV is scarce. The STREAM trial, which showed that the STR was noninferior to long MDR/RR-TB treatment regimen (LTR) in terms of treatment success, included only 80 HIV-coinfected patients and remained inconclusive with regard to the effectiveness of the STR in this subgroup.^8^ Another study evaluating the STR in nine countries showed that mortality was higher among HIV-coinfected patients (19% versus 5% in HIV-negative) but did not compare the effectiveness of the STR with the LTR.^6^ Moreover, it was conducted in settings with low HIV prevalence and did not assess predictors of mortality among HIV-coinfected patients treated for MDR/RR-TB.^6^ How to reduce mortality in HIV-coinfected patients treated for MDR/RR-TB remains unknown. Therefore, we first estimated the effect of HIV-coinfection on mortality among all patients started on MDR/RR-TB treatment between 2005 and 2018 in the Rwandan PMDT. Second, we estimated the effect of using the STR versus the LTR and assessed other predictors of mortality among HIV-coinfected patients treated for MDR/RR-TB.

## METHODS

### Design and study population.

We conducted a retrospective cohort analysis that included all patients treated for MDR/RR-TB between July 2005 and December 2018 in Rwanda.

### Study settings and organization of MDR/RR-TB care.

This study was conducted in Rwanda, a central eastern Africa country with ∼13 million people in 2019. The estimated TB burden in Rwanda was 57 per 100,000 persons in 2019. Overall, TB prevalence in Rwanda has been declining since 2006.^5^ The latest estimated rate of RR was relatively low (2.2% in new and 7.1% in previously treated TB patients) compared with the previous estimate^1^. Over the years, RR testing has been expanded to most TB patients (e.g., 92% in new and 89% in previously treated TB patients in 2019). Since 2014, patients with sputum smear-positive TB were systematically tested for MDR/RR using the Xpert MTB/RIF assay. In those presenting with presumptive TB and living with HIV, the Xpert MTB/RIF assay was used as the first test for TB diagnosis because it has better sensitivity than smear microscopy. In the years before Xpert MTB/RIF implementation, susceptibility to rifampicin and other drugs was tested with phenotypic or line probe (Hain Lifescience, Nehren, Germany) assays. When MDR/RR-TB was identified, patients were admitted in one of the three national MDR/RR-TB treatment centers for hospitalization and directly observed treatment until culture conversion and improved clinical status. For treatment follow-up, a monthly culture and biweekly sputum smear microscopy were done. All health facilities provided integrated TB and HIV services. HIV testing was offered to all presumptive TB patients if HIV status was unknown. For HIV-coinfected MDR/RR-TB patients, antiretroviral therapy (ART) was prescribed by their MDR/RR-TB care providers.

### MDR/RR-TB regimen.

The LTR was implemented starting in July 2005 and comprised an intensive phase of 6 to 8 months, followed by a continuation phase of 14 months (6–8 Km-Lfx-Pto-Cs-Z/14Lfx-Pto-Cs-[Z]). The STR was implemented in July 2014 as part of a multicountry research project coordinated by the International Union Against Tuberculosis and Lung Disease and comprised an intensive phase of 4 to 6 months, followed by a continuation phase of 5 months (4–6 Km-Mfx-Pto-Cfz-H-E-Z/5Mfx-Cfz-E-Z).

### Data collection.

As described previously,^4^ the data were collected from patients’ files, the National Reference Laboratory, and National MDR/RR-TB Program databases. For the present analysis, we included also the data from the 2017 and 2018 cohorts. We collected additional data on HIV-related variables—namely, the use of ART at TB treatment initiation (yes or no), ART regimen, CD4 cell count at ART initiation, and the latest CD4 cell count. Data entry was carried out using EpiData database (EpiData Association, Odense, Denmark). The CD4 at TB treatment initiation was the CD4 cell count with a date closest to the date of MDR/RR-TB treatment initiation but not more than 365 days apart.

### Data analysis.

STATA v.16.2 (Stata Corp, College Station, TX) was used for data analysis. The χ^2^ and Fisher’s exact tests were used to assess the association between categorical variables. Bivariable logistic regression was used to estimate the association between independent variables and death. Variables associated at the level of 0.2 were included in a multivariable regression, together with treatment regimen and year of TB treatment initiation. This multivariable model was simplified until it contained only those variables associated at the level of 0.05 plus treatment regimen and year of TB treatment regimen. For some categories with rare events, we used Firth’s logistic regression. The same approach was used to assess predictors of mortality in HIV-coinfected treated for MDR/RR-TB. As a sensitivity analysis, we calculated predictors of having a programmatically adverse outcome by adding those lost to follow-up or experiencing treatment failure, to those who died. We estimated the detectable effect size with regard to the difference in mortality for using either the STR or LTR. Statistical significance was set at 0.05.

### Ethics.

The Rwanda National Ethical Committee ratified the study protocol with reference number IRB 00001497 of IORG0001100; Ref No. 0069/RNEC/2017). In addition, the Institutional Review Board of the Institute of Tropical Medicine, Antwerp, Belgium, with reference number IRB/AB/AC/062; Ref No. 1208/17; 19/03/2018) together with the Antwerp (Belgium) University Hospital Ethics Committee (UZA, Universitair Ziekenhuis Antwerpen Ethische Commissie), with REG No. B300201836458; 14/05/2018; approved the study protocol.

## RESULTS

**Figure 1.**
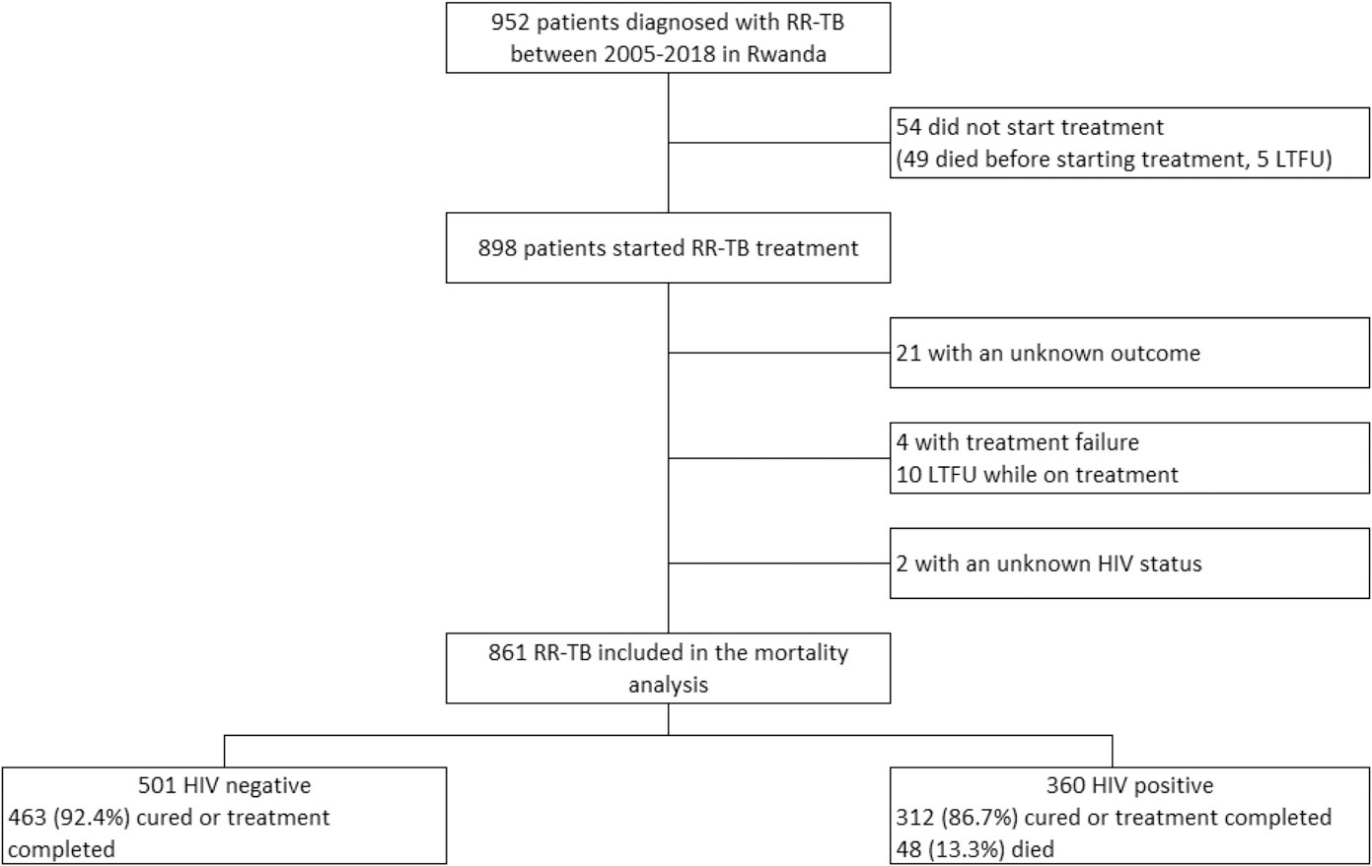
Enrollment among patients diagnosed with rifampicin-resistant tuberculosis in Rwanda.

Among 861 (95.9%) patients included in the mortality analysis, 360 (41.8%) were HIV-coinfected (Table 1). Among HIV-negative patients, 92.4% had a favorable outcome and 7.6% died, whereas among HIV-coinfected patients, 86.7% had a favorable outcome and 13.3% died (Figure 1 and Table 2).

**Table 1:** Baseline characteristics by HIV status among 861 patients treated for multidrug-resistant/rifampicin-resistant tuberculosis in Rwanda

	Total		HIV negative		HIV-positive		
	*N*	%	*n*	%	*n*	%	*P* value
Total	861		501		360		NA
Gender							0.002
Female	347	40.3	180	35.9	167	46.4	
Male	514	59.7	321	64.1	193	53.6	
Age group							< 0.001
< 29	290	33.7	207	41.3	83	23.1	
30–44	343	39.8	150	29.9	193	53.6	
45–54	121	14.1	62	12.4	59	16.4	
≥ 55	104	12.1	80	16	24	6.7	
Missing	3	0.3	2	0.4	1	0.3	
Treatment history							0.7
No previous treatment	300	34.8	168	33.5	132	36.7	
One first-line treatment	238	27.6	146	29.1	92	25.6	
Two first-line treatments	298	34.6	174	34.7	124	34.4	
Second-line treatment	7	0.8	4	0.8	3	0.8	
Missing	18	2.1	9	1.8	9	2.5	
Treatment regimen							0.6
Short regimen	333	38.7	190	37.9	143	39.7	
Long regimen	528	61.3	311	62.1	217	60.3	

NA = not applicable.

**Table 2:** Predictors of mortality among 861 patients treated for multidrug-resistant/rifampicin-resistant tuberculosis in Rwanda.

	Total	Died					
	*N*	*n*	%	OR	95% CI	aOR†	95% CI
Total	861	86	10.0	NA		NA	
Gender						NS	
Female	347	29	8.4	1			
Male	514	57	11.1	1.37	0.86–2.19		
Age group							
< 29	290	18	6.2	1		1	
30–44	343	34	9.9	1.66	0.92–3.01	1.37	0.74–2.56
45–54	121	15	12.4	2.14*	**1.04–4.40**	1.89	0.89–4.01
≥ 55	104	19	18.3	3.38***	**1.70–6.73**	3.79***	**1.83–7.85**
Treatment history							
No previous treatment	300	26	8.7	1		1	
One first-line treatment	238	21	8.8	1.02	0.56–1.86	1.17	0.61–2.24
Two first-line treatments	298	31	10.4	1.22	0.71–2.12	1.55	0.74–3.25
Second-line treatment	7	3	42.9	7.90**	**1.68–37.24**	9.54**	**1.85–49.26**
Treatment regimen							
Short regimen	333	34	10.2	1		1	
Long regimen	528	52	9.8	0.96	0.61–1.52	1.14	0.49–2.69
HIV status							
Negative	501	38	7.6	1		1	
Positive	360	48	13.3	1.87**	**1.20–2.94**	2.05**	**1.26–3.33**

Missing data not shown; for age group, data were missing for three patients; for treatment history, data were missing for 18 patients. Bold data shows variables which have remained significantly different after bivariable and multivariable analysis in all patients treated for MDR/RR-TB regardless of HIV status. aOR = adjusted odds ratio; CI = confidence interval; NA = not applicable; NS = not significant; OR = odds ratio.

* *P* < 0.05; ** *P* < 0.01; *** *P* < 0.001.

† Adjusted for variables shown and year of tuberculosis treatment start.

All four patients who had treatment failure were HIV-coinfected. Considering only cure and treatment failure as outcomes, failure was more frequent among HIV-coinfected patients (1.1% (4/360) versus 0.0% (0/463); *P* = 0.03). Two of 10 patients lost to follow-up were HIV-coinfected, whereas three of the 10 patients were treated with the STR. Three of four patients with treatment failure were treated with the LTR.

### Patient characteristics.

Among HIV-coinfected patients, compared with HIV-negative patients, a higher proportion was female (46.4% versus 35.9%, *P* = 0.002; Table 1). Among HIV-coinfected patients, a larger proportion belonged to 30- to 44-year age group (53.6% versus 29.9%), and a smaller proportion belonged to the 55-year and older age group (6.7% versus 16.0%). The treatment history was similar between HIV-coinfected and HIV-negative patients: 300 (34.8%) were new TB patients, 238 (27.6%) had one previous first-line treatment, 298 (34.6%) had at least two previous first-line treatments, and 7 (0.8%) were treated for the second-time for MDR/RR-TB. Overall, 333 (38.7%) were treated with the STR, with a similar proportion among HIV-coinfected and HIV-negative patients (Table 1).

In a sensitivity analysis, in which lost to follow-up, treatment failure, and death were considered as adverse outcomes, being 55 years or older (adjusted odds ratio [aOR] 2.68; 95% CI: 1.36–5.26), previous second-line treatment (aOR 7.25; 95% CI: 1.44–36.39), and being HIV-coinfected (aOR 1.86; 95% CI: 1.19–2.91) were independently correlated with having this composite adverse outcome, whereas being treated with an LTR was not (aOR 1.14; 95% CI: 0.51–2.54).

### Characteristics of HIV-coinfected MDR/RR-TB patients.

Of 360 HIV-coinfected patients, 143 (39.7%) were treated with the STR (Table 3). A higher proportion was male among patients treated with the STR (65.7% versus 45.6% with the LTR; *P* < 0.001). The proportion of patients aged 55 years or older was higher among those treated with the STR (11.9% versus 3.2%). The proportion of previously treated patients was significantly higher among those treated with the LTR (83.4% versus 28.7%).

**Table 3:** Characteristics by regimen among 360 PLHIV treated for multidrug-resistant/rifampicin-resistant tuberculosis in Rwanda

	Total		Short regimen		Long regimen		
	*N*	%	*n*	%	*n*	%	*P* value
Total	360		143		217		NA
Gender							< 0.001
Female	167	46.4	49	34.3	118	54.4	
Male	193	53.6	94	65.7	99	45.6	
Age group							0.01
< 29	83	23.1	27	18.9	56	25.8	
30–44	193	53.6	74	51.7	119	54.8	
45–54	59	16.4	24	16.8	35	16.1	
≥ 55	24	6.7	17	11.9	7	3.2	
Missing	1	0.3	1	0.7	0	0	
TB treatment history							< 0.001
No previous treatment	132	36.7	99	69.2	33	15.2	
Previously treated	222	61.7	41	28.7	181	83.4	
Missing	6	1.7	3	2.1	3	1.4	
CD4 at TB treatment initiation							0.1
>500	46	12.8	17	11.9	29	13.4	
>200–500	113	31.4	36	25.2	77	35.5	
> 100–200	30	8.3	14	9.8	16	7.4	
≤ 100	46	12.8	25	17.5	21	9.7	
Missing	125	34.7	51	35.7	74	34.1	
ART when starting TB treatment							0.5
ART before TB treatment start	218	60.6	92	64.3	126	58.1	
ART after TB treatment start	74	20.6	26	18.2	48	22.1	
ART initiation not reported	68	18.9	25	17.5	43	19.8	
ART regimen							< 0.001
D4T/3TC/NNRTI	63	17.5	0	0	63	29	
TDF/3TC/NNRTI	153	42.5	82	57.3	71	32.7	
ABC/3TC/NNRTI	18	5	10	7	8	3.7	
AZT/3TC/NNRTI	34	9.4	6	4.2	28	12.9	
PI-based	3	0.8	3	2.1	0	0	
ART regimen unknown	21	5.8	17	11.9	4	1.8	
ART initiation not reported	68	18.9	25	17.5	43	19.8	
Outcome							0.5
Cure or treatment completed	312	86.7	122	85.3	190	87.6	
Died	48	13.3	21	14.7	27	12.4	

3 TC = lamivudine; ABC = abacavir; ART = antiretroviral therapy; AZT = zidovudine; D4T = stavudine; NA = not applicable; NNRTI = nonnucleoside reverse transcriptase inhibitor; PI = protease-inhibitor; PLHIV = people living with HIV; TB = tuberculosis; TDF = tenofovir.

For 125 (34.7% of 360) HIV-coinfected patients, the CD4 count at MDR/RR-TB initiation was unknown. The CD4 count was higher than 500 cells/mm^3^ for 12.8%, one-third (31.4%) had a CD4 count between 200 and 500 cells/mm^3^, 8.3% had CD4 count ranging between 100 to 200 cells/mm^3^, and 12.8% had a CD4 lower than 100 cells/mm^3^ (distribution was similar in both the LTR and STR groups). Moreover, 60.6% of the patients were on ART before MDR/RR-TB treatment was started, whereas 20.6% were started on ART during MDR/RR-TB treatment. The timing of ART initiation was not specified for 68 (18.9%) patients. The most frequently used ART regimen was TDF/3TC plus a nonnucleoside reverse transcriptase inhibitor. For 21 (5.8%) patients, the ART regimen was unknown. The overall mortality among HIV-coinfected patients was 13.3%.

### Predictors of mortality.

In total, 86 (10.0%) patients died during treatment (Table 2). After adjustment for type of regimen and year of MDR/RR-TB initiation, with all variables included in the multivariable model, patients aged 55 years and older (aOR 3.79; 95% CI: 1.83–7.85), patients previously exposed to second-line TB treatment (aOR 9.54; 95% CI: 1.85–49.26), and those who were HIV-coinfected (aOR 2.05; 95% CI: 1.26–3.33) had a higher mortality (Table 4). Mortality in HIV-coinfected patients with a CD4 > 200 cell/mm^3^ was not different from HIV-negative patients (10.1% [16/159] versus 7.6% [38/501]; aOR 1.42; 95% CI: 0.73–2.75). The detectable effect size with regard to the difference in mortality for using either the STR or LTR was 6.5% (16.3% mortality in the STR cohort would have been significantly different, given 9.8% mortality in the LTR group, *N* = 861, power 0.8, alpha 0.05, and ratio STR/LTR 0.65).

**Table 4:** Predictors of mortality among 360 PLHIV treated for multidrug-resistant/rifampicin-resistant tuberculosis in Rwanda.

	Total	Died					
	N	N	%	OR	95%CI	aOR‡	95% CI
Total	360	48	13.3				
Gender						NS	
Female	167	15	9	1			
Male	193	33	17.1	2.09*	**1.09–4.00**		
Age group							
< 29	83	6	7.2	1		1	
30–44	193	27	14	1.97	0.80–4.83	1.98	0.80–4.91
45–54	59	8	13.6	1.97	0.67–5.80	1.84	0.61–5.50
≥ 55	24	7	29.2	5.11**	**1.58–16.49**	5.89**	**1.73–20.05**
TB treatment history						NS	
No previous treatment	132	16	12.1	1			
Previously treated	222	30	13.5	1.13	0.59–2.17		
CD4 at TB treatment initiation							
> 500	46	4	8.7	1		1	
> 200–500	113	12	10.6	1.25	0.38–4.09	1.36	0.43–4.33
> 100–200	30	3	10	1.17	0.24–5.63	0.98	0.22–4.43
≤ 100	46	12	26.1	3.71*	**1.10–12.53**	3.77*	**1.13–12.58**
ART when starting TB treatment						NS	
ART before TB treatment	218	27	12.4	1			
ART after TB treatment	74	8	10.8	0.86	0.37–1.98		
ART initiation not reported	68	13	19.1	1.67	0.81–3.46		
ART regimen†						NS	
D4T/3TC/NNRTI	63	3	4.8	1			
TDF/3TC/NNRTI	153	21	13.7	2.8	0.87–9.04		
ABC/3TC/NNRTI	18	4	22.2	5.36*	**1.19–24.28**		
AZT/3TC/NNRTI	34	5	14.7	3.22	0.79–13.21		
PI-based	3	1	33.3	10.37*	**1.05–102.74**		
Regimen							
Short	143	21	14.7	1		1	
Long	217	27	12.4	0.83	0.45–1.53	2.13	0.80–5.65

Age group data were missing for one patient; treatment history data missing for six patients; CD4 data missing for 125 patients; ART regimen was unknown for 21 patients. Bold data shows variables which have remained significantly different after bivariable and multivariable analysis in People living with HIV only. 3TC = lamivudine; ABC = abacavir; ART = antiretroviral therapy; AZT = zidovudine; D4T = stavudine; NA = not applicable; NNRTI = nonnucleoside reverse transcriptase inhibitor; NS = not significant; PI = protease-inhibitor; PLHIV = people living with HIV; TB = tuberculosis; TDF = tenofovir.

* *P* < 0.05; **P < 0.01; ***P < 0.001.

† Category for those not on ART not shown.

‡ Adjusted for variables shown and year of TB treatment start.

For HIV-coinfected patients, after adjustment for treatment regimen and year of MDR/RR-TB initiation, and all variables included in the multivariable model, age 55 years or older (versus those 29 years old or younger; aOR 5.89; 95% CI: 1.73–20.05), those with a CD4 count of 100 cells/mm^3^ or less at MDR/RR-TB initiation (versus those with a CD4 count higher than 500 cells/mm^3^; aOR 3.77; 95% CI: 1.13–12.58) were more likely to die. The type of regimen (STR versus LTR) was not correlated with mortality (Table 4). Considering loss to follow-up, treatment failure, and death as adverse outcomes, the sensitivity analysis showed that age 55 years or older (aOR 4.24; 95% CI: 1.34–13.43) was correlated with having this composite adverse outcome, whereas CD4 < 100 cells/mm^3^ at TB treatment start (aOR 2.98; 95% CI: 0.97–9.16) and being treated with an LTR was not (aOR 1.98; 95% CI: 0.77–5.08).

## DISCUSSION

This analysis of a nationwide MDR/RR-TB dataset spanning 14 years demonstrates patient outcomes and predictors of mortality among HIV-coinfected patients in a programmatic context. Overall, HIV coinfection, patients aged 55 years and above, and patients previously exposed to second-line TB treatment had a higher mortality. Among HIV-coinfected patients, the mortality was not statistically different for those treated with STR compared with those treated with LTR. Among HIV-coinfected patients, those aged 55 years and older, those with a baseline CD4 count ≤ 100 cells/mm^3^ were more at risk of dying. However, the mortality among HIV-coinfected patients with a CD4 count higher than 200 cells/mm^3^ was not statistically different from HIV-negative patients.

The correlation between the immunosuppression and mortality is not surprising because TB treatment outcomes depend on host immunity.^9^ Studies have shown a key role of acquired cellular immunity, led by CD4, in fighting reactivation of TB infection.^10^ This implies the need for close CD4 cell count monitoring for this specific group. Data from African settings showed that immunosuppressed HIV-coinfected patients were more likely to die from opportunistic infections including tuberculosis.^11^ More than 50% of HIV/AIDS-related deaths result from tuberculosis and other infections, such as cryptococcal meningitis and severe bacterial infections.^12^

ART initiation in an early stage of HIV disease, regardless of the CD4 cell count, greatly protects patients.^13^ However, in our analysis, there was no difference in mortality for those who started ART before compared with after initiation of an MDR/RR-TB regimen. This contrasts with findings from an individual patient data meta-analysis, which clearly showed that the odds of dying was associated with not using ART at the time of MDR/RR-TB treatment initiation^14^: In our study, the number of patients who started ART after starting MDR/RR-TB treatment was too small to identify a correlation. Since 2016, all HIV-coinfected patients have been started on ART irrespective of the CD4 count. Future studies should explore whether the “Treat All” guidelines indeed result in reduced MDR/RR-TB mortality in HIV-coinfected patients.

In our study, the mortality among HIV-coinfected patients treated with either the STR or the LTR was not statistically different. Our findings complement those of the STREAM trial, which was not powered to assess the noninferiority of the STR in HIV-coinfected patients.^7^ A meta-analysis showed that the STR less frequently resulted in loss to follow-up.^17^ This meta-analysis also showed that the relapse and failure rates were higher for the STR.^17^ In our study, few patients who experienced treatment failure were treated with the LTR, but we do not provide data on the relapse rate. Moreover, the comparison between the STR and the LTR may have been affected by the before–after design. For instance, diagnostic and therapeutic delay reduced as rapid molecular drug susceptibility testing (DST) was implemented.^4^

Consistent with other studies,^15^ older age was a strong predictor of mortality both overall and among HIV-coinfected patients. This might be related to higher risk of other comorbidities, such as metabolic diseases, immunosenescence, other comorbidities, and drug interactions.^16,17^

Also consistent with previous studies,^4,18^ the history of exposure to second-line TB treatment was independently associated with mortality. This might be due to the accumulation of resistance. Although resistance to fluoroquinolone, the second-line core drug, fortunately remains rare in Rwanda,^19^ full DST at the start of the second-line treatment as well as a close follow-up, including DST during treatment of late positive cultures, is justified.

Strengths of our study include the comprehensive analysis of most PLHIV diagnosed with MDR/RR-TB who started MDR/RR-TB treatment since the beginning of PMDT in 2005 through December 2018. Moreover, different sources, such as the national reference laboratory files, patient files, and PMDT national registers were used to resolve discrepancies or missing data. Nevertheless, the study has some limitations. A large proportion of HIV-coinfected patients (34.7%) had no data on CD4 count or timing of ART initiation, and we did not have viral load data. No data are shown for comorbidities beyond HIV coinfection or drug-related adverse events. Data on the comparison between STR and LTR outcomes reflect the reality of the program, which changed over time. Therefore, this comparison may have been affected by selection bias, as diagnostic and therapeutic delay decreased over time, as did pretreatment mortality and lost to follow-up. Patients incorrectly enrolled on MDR-TB treatment due to false RR-TB on Xpert MTB/RIF assay^20^ were not excluded from analysis. However, the occurrence of false RR was not associated with predictors of mortality described in this study. Finally, the two types of MDR-TB regimens assessed in this study contained a second-line injectable. On the basis of observational data from a single study, the WHO recommends using all-oral regimens.^21^ When Rwanda implements all-oral RR-TB treatment, we will also evaluate its effect on outcomes, including acquired resistance and results of salvage regimens for those with an unfavorable outcome.

In conclusion, advanced HIV/AIDS status remains the major predictor of mortality in HIV-coinfected MDR/RR-TB patients. Even though CD4 count monitoring is no longer done routinely in PLHIV, we recommend systematically determining the baseline CD4 before starting MDR/RR-TB treatment and providing advanced HIV care to patients with a CD4 below 200 cell/mm^3^. PMDT should put in place a separate MDR/RR-TB management guidelines for elderly with special consideration for elderly with comorbidities. In HIV-coinfected patients, the STR was as effective as the LTR. Therefore, the use of the STR is highly recommended because of the benefits associated with a shorter treatment duration. Moreover, drug susceptibility testing is highly recommended for MDR/RR-TB patients who have been previously exposed to second-line treatment to rule out any resistance at the start of a second round of MDR/RR-TB treatment, preferably with results available at least within the first month. Fortunately, resistance to fluoroquinolones, the core drug class for second-line treatment, remains low in Rwanda, and genotypic DST is available with Line Probe Assays. Future studies that will systematically characterize comorbidities encountered in older MDR/RR-TB patients are needed. In addition, future studies on a suppressed viral load as a current reference standard for HIV treatment success would elucidate more insight on predictors of MDR/RR-TB mortality among HIV-positive patients.
